# Supplementary material for: Recent Evolution in Rattus norvegicus Is Shaped by Declining Effective Population Size
Source: Mol Biol Evol. 2015 Jun 1;32(10):2547–58. doi: 10.1093/molbev/msv126 (PMC4576703; doi:10.1093/molbev/msv126)
Supplement: Supplementary Data [file supp_32_10_2547__index.html]

Supplementary Data 

# Recent Evolution in *Rattus norvegicus* Is Shaped by Declining Effective Population Size

## Supplementary Data

files

- Supplementary Data - pdf file
