# Supplementary material for: Recent Evolution in Rattus norvegicus Is Shaped by Declining Effective Population Size
Source: Mol Biol Evol. 2015 Jun 1;32(10):2547–58. doi: 10.1093/molbev/msv126 (PMC4576703; doi:10.1093/molbev/msv126)
Supplement: Supplementary Data [file supp_msv126_ratDiversity_supplementary_alone.pdf]

Recent evolution in *Rattus norvegicus* is shaped by declining effective population size.

Supplementary material

Authors: Eva E. Deinum[1], Daniel L. Halligan[1], Rob W. Ness[1], Yao-Hua Zhang[2], Lin Cong[3], Jian-Xu Zhang[2], and Peter D. Keightley[1]*

[1]: Institute of Evolutionary Biology, University of Edinburgh, Edinburgh, United Kingdom
[2]: State Key Laboratory of Integrated Management of Pest Insects and Rodents in Agriculture, Institute of Zoology, Chinese Academy of Sciences, 1# Bei-Chen West Road, Beijing 100101, China
[3]: Institute of Plant Protection, Heilongjiang Academy of Agricultural Sciences, Harbin 150086, China

*: Corresponding author:
Institute of Evolutionary Biology
University of Edinburgh
West Mains Rd
Edinburgh EH9 3FL
UK
peter.keightley@ed.ac.uk

1 Bootstrapping for exons

To obtain confidence intervals of genome wide (average) quantities we applied bootstrapping by sampling with replacement among the annotated transcripts. With this procedure, sites in overlapping transcripts would on average be overrepresented in the bootstrapping sets. We therefore initially used several sets of transcripts for bootstrapping: "All" – all transcripts; "NoOv" – all non-overlapping transcripts; "FuNoOv" – all transcripts with a functional annotation in the EggNog v4 database OR non-overlapping. The rationale behind the FuNoOv set is the observation that only one of the overlapping transcripts appears in the database. We also used sets "All\X" (identical to "FuNoOv\X") and "NoOv\X" excluding the non-annotated transcripts from the respective sets. The statistics obtained when accounting for all positions exactly once were most similar to the NoOv set when considering all exonic sites and most similar to All\X when excluding sites that only appeared in non-annotated transcripts, so we used NoOv and All\X for bootstrapping. We further observed that the size of the 95% confidence intervals was similar for all sets and all were +/- centered around the sample average and median (figs. S1, S2, S3). We therefore used the bootstrapping sets to obtain the size of the error bars relative to the median of the bootstrapping samples and added those to the plain values in fig. 1 and table S1.

2 Estimating a lower bound for $\langle r^2 \rangle$

To calculate r^2 between a pair of biallelic sites, the sites were first converted into vectors consisting of 0 (homozygote reference), 1 (heterozygote) and 2 (homozygote alternate). For a small sample size (such as 10 or 12 individuals as in our case), the average r^2 will be significantly higher than 0 for completely random vectors. For a combination of two singletons this is $\frac{1}{n-1}$: in $\frac{1}{n}$ of the cases, $r^2 = 1$, and in $\frac{n-1}{n}$ of the cases $r^2 = \left(\frac{1}{n-1}\right)^2$, which adds up to $\frac{1}{n-1}$. For haplotype based correlation measures of LD, the range and expected value of r do not depend on minor allele frequency. To test if this would hold for r^2, we generated 300, 1000 and 3000 random genotype vectors of sample size length for each minor allele frequency. We then calculated pairwise r^2 for each pair of random vectors with a particular combination of minor allele frequencies (e.g. 1/24 with 3/24 for a sample of $n = 12$ diploid individuals). All values converged to $\frac{1}{n-1}$ with increasing numbers of random vectors (data not shown). This is similar to Weir's correction for sample size of $\hat{\Delta} = \Delta \frac{n}{n-1}$ (Weir, 1979).

3 Robustness of PSMC inference

PSMC inference is based on the length distribution of stretches of sequence that are homozygous or "identical by state" (IBS). Running the PSMC analysis is computationally costly, so we first determined which filter had the largest impact on the normalized distributions of homozygous runs (HHn MacLeod et al. (2013)). HHn is defined as the actual number of IBS stretches of a particular length divided by the number that could maximally occur in the data.

In the definition of HHn in MacLeod et al. (2013), it is not specified how to handle gaps in the data. We introduced a maximum gap length parameter (g for the remainder of this text, with default g=100). For larger gaps in the data, the sequences on either side of the gap are treated as on independent molecules. We used the first 1 Mb of chromosome 6 as a test set for this screen.

The filter that had by far the strongest impact on the HHn curves was the proximity filter when varied over a range of md=0, (disabled) md=5 (default) and md=10 (overly aggressive) fig. S12). This filter also had the strongest impact on overall heterozygosity.

We therefore repeated the PSMC inference with the 3 different cutoffs (fig. S11). As expected, estimates of the most recent N_e estimate were most sensitive to this filter, because the longest stretches of homozygosity are most likely to be broken up by residual false positive heterozygote sites MacLeod et al. (2013). We also observed a decrease of the estimated ancient N_e. This trend is also an anticipated effect of the proximity filter, as it specifically removes the shortest runs of homozygosity. Importantly, all graphs consistently show a historic decrease of N_e, ranging from almost 4-fold (md=0) to over 2-fold (md=10).

A note on parameter g: this is not an explicit parameter to PSMC, so within this screening context the only relevant aspect is that it should not disturb the analysis too much. fig. S12B shows that this is the case: where g was not so small that it became disruptive (e.g., g=10 with md=10), it had a smaller impact than varying the minimum GQ (fig. S12A). For a serious analysis along the lines of MacLeod et al. (2013), a value of $g \approx$ 1/heterozygosity seems reasonable, or an approach of drawing random heterozygous sites, proportional to overall heterozygosity, within the missing sequence and "breaking" the molecule only for "very large" gaps.

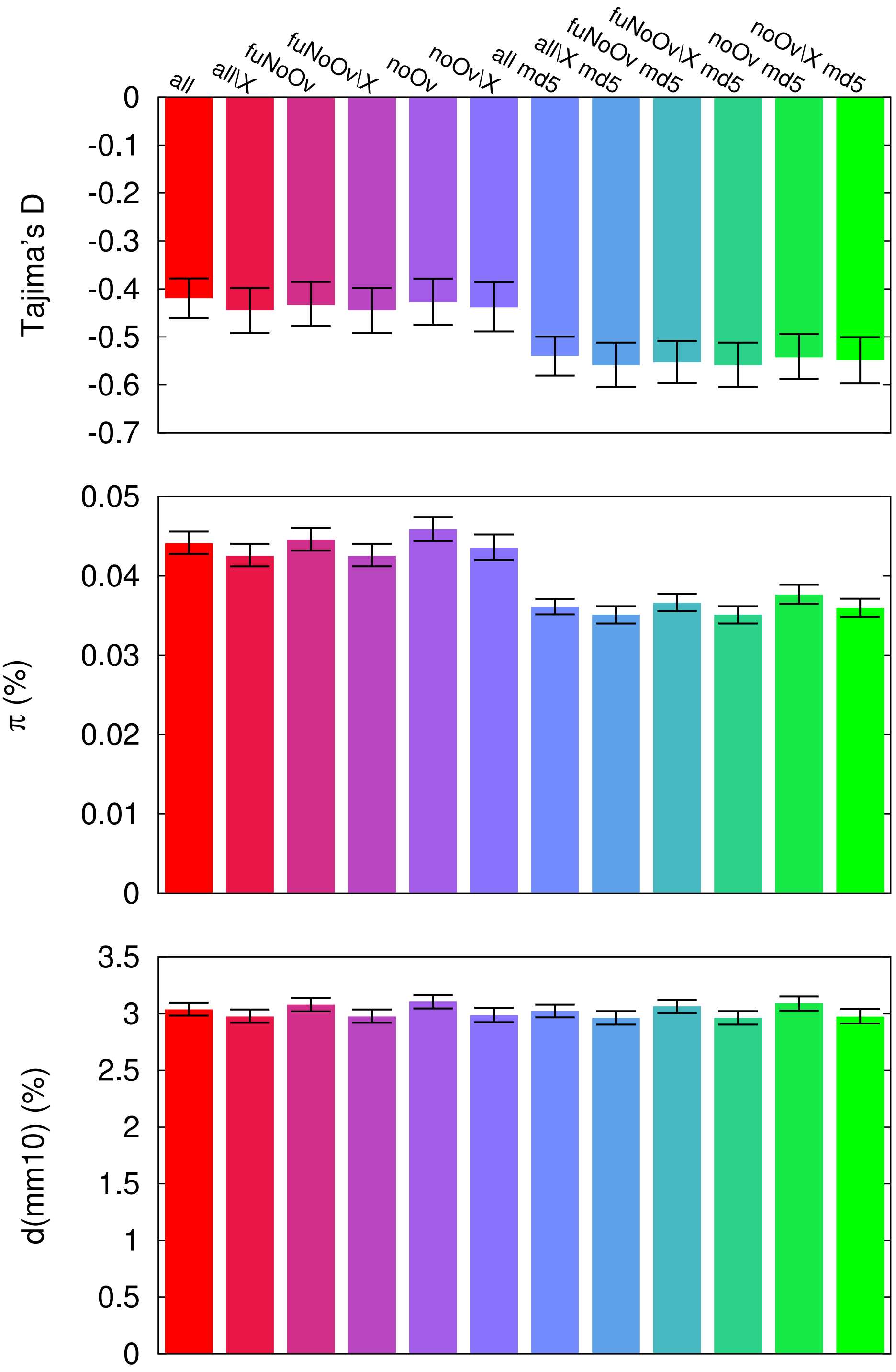

Figure S1: Impact of proximity filter and transcript selection rules (for bootstrapping analysis) on Tajima's D, diversity and divergence from mouse for wild *R. norvegicus* data for 0-fold degenerate exonic sites. Error bars show 95% confidence intervals based on 1000 bootstrapping replicates. Labels: all: all transcripts; noOv: only non-overlapping transcripts; fuNoOv: all transcripts with a function assigned (eggNog v4 database) OR non-overlapping; \ X: excluding transcripts without function assignment; md5: application of the proximity filter with default md = 5 bp. Note that the proximity filter has very little impact on divergence, but has a much stronger impact than the transcript selection rules on the other statistics.

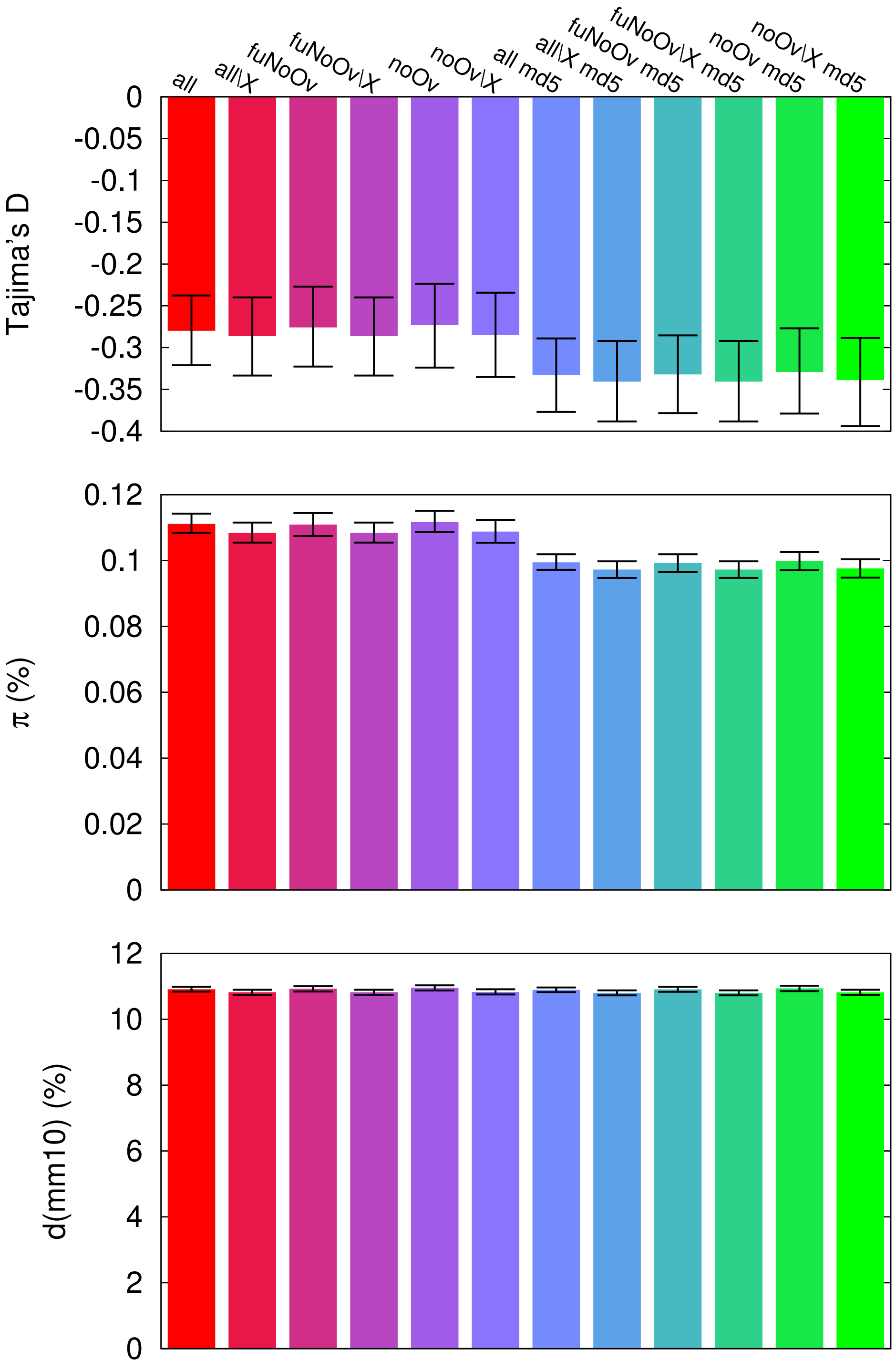

Figure S2: Impact of proximity filter and transcript selection rules (for bootstrapping analysis) on Tajima's D, diversity and divergence from mouse for wild *R. norvegicus* data for 2-fold degenerate exonic sites. Error bars show 95% confidence intervals based on 1000 bootstrapping replicates. Labels: all: all transcripts; noOv: only non-overlapping transcripts; fuNoOv: all transcripts with a function assigned (eggNog v4 database) OR non-overlapping; $\setminus$ X: excluding transcripts without function assignment; md5: application of the proximity filter with default md = 5 bp. Note that the proximity filter has very little impact on divergence, but has a much stronger impact than the transcript selection rules on the other statistics.

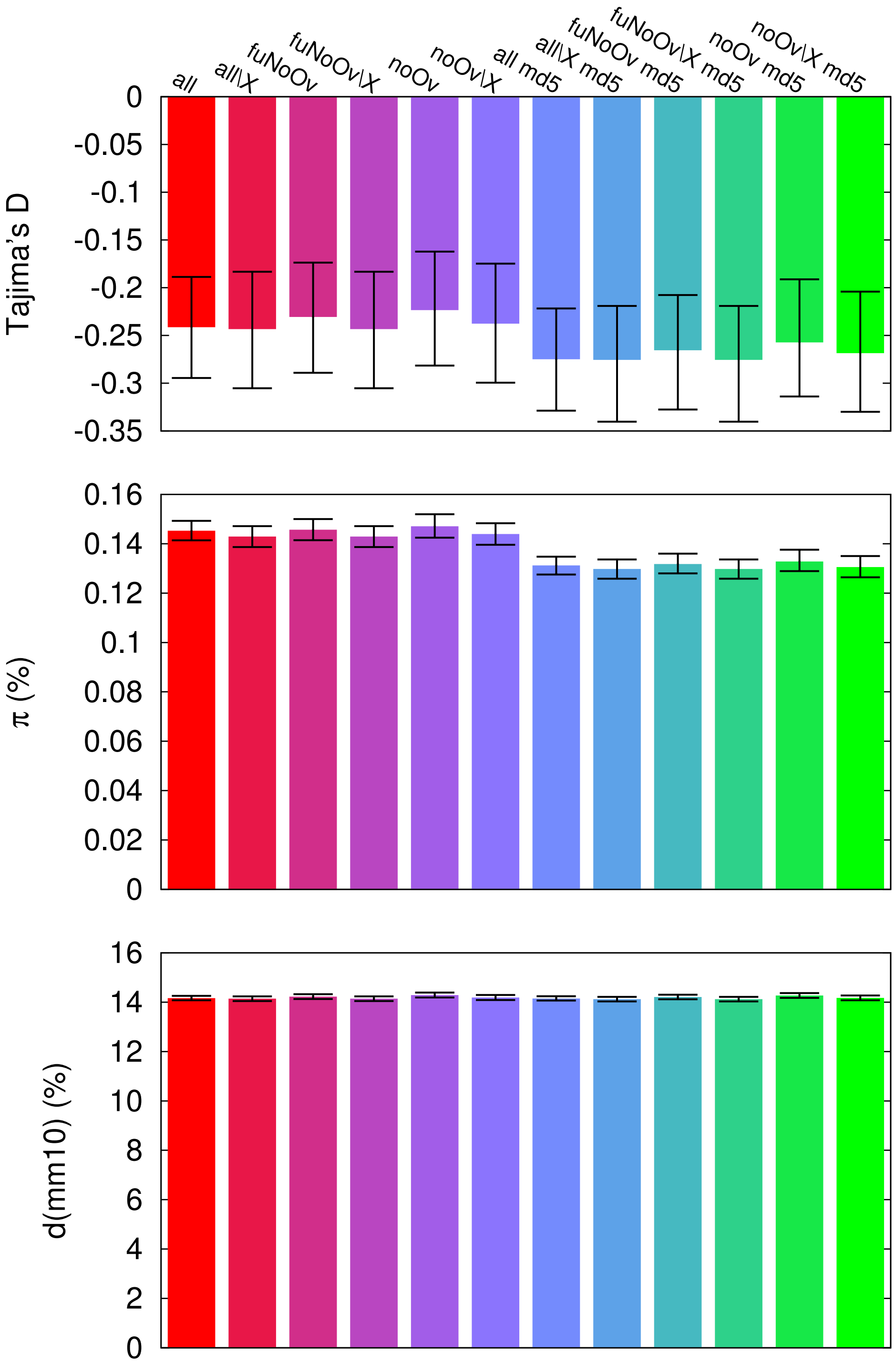

Figure S3: Impact of proximity filter and transcript selection rules (for bootstrapping analysis) on Tajima's D, diversity and divergence from mouse for wild *R. norvegicus* data for 4-fold degenerate exonic sites. Error bars show 95% confidence intervals based on 1000 bootstrapping replicates. Labels: all: all transcripts; noOv: only non-overlapping transcripts; fuNoOv: all transcripts with a function assigned (eggNog v4 database) OR non-overlapping; $\setminus$ X: excluding transcripts without function assignment; md5: application of the proximity filter with default md = 5 bp. Note that the proximity filter has very little impact on divergence, but has a much stronger impact than the transcript selection rules on the other statistics.

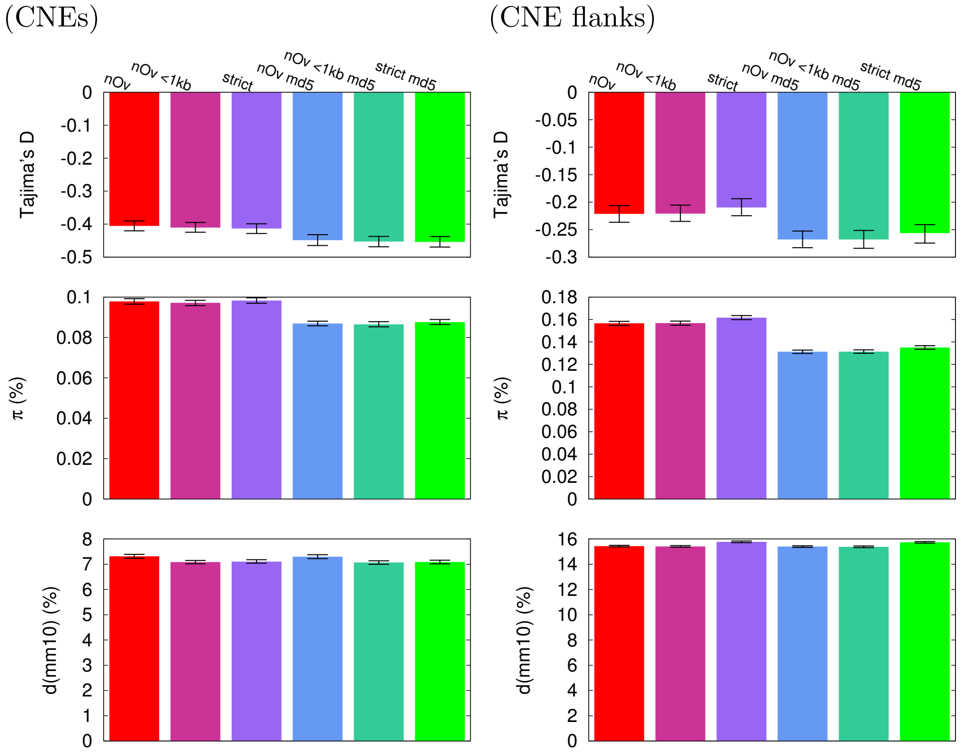

Figure S4: Impact of filters on Tajima's D, diversity and divergence from mouse for wild *R. norvegicus* data. Error bars show 95% confidence intervals based on 1000 bootstrapping replicates. Labels: nOv: "no Overlap" = excise exonic sequence from CNE candidate locations; nOv <1kb: "no Overlap" restricted to elements of <1 kb length; strict: completely discard all CNE candidates with any overlap with exons; md5: application of the proximity filter with default md = 5 bp. Note that the proximity filter has very little impact on divergence, but has a much stronger impact than the CNE selection rules on the other statistics.

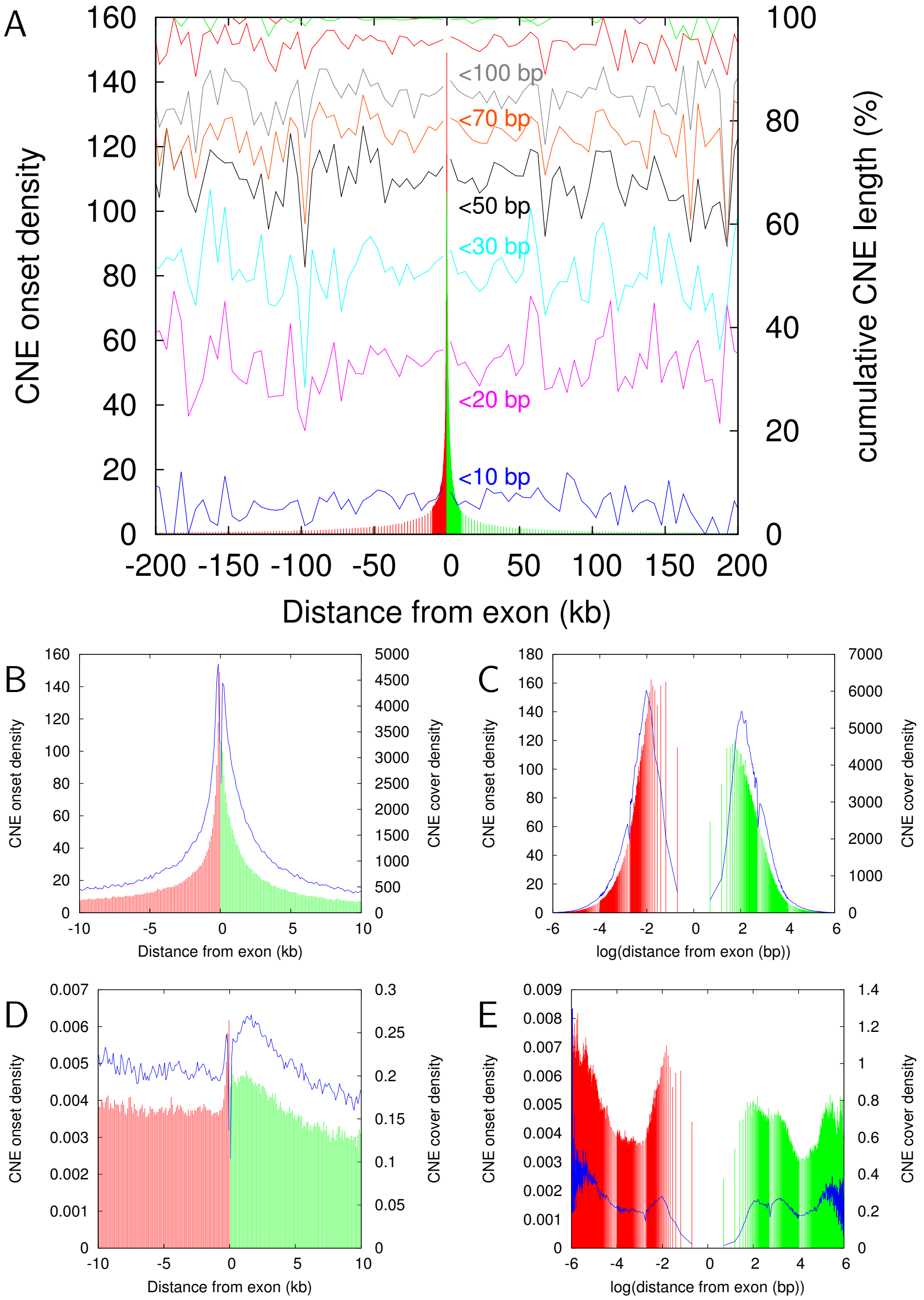

Figure S5: CNE location – Distribution of CNE distances to nearest exon and density of CNE "cover" with distance to nearest exon. The ratio of short to medium length CNEs does not depend on distance to the nearest exon (A). The noisy lines indicate the cumulative fraction of CNEs at given location with lengths < 10 (blue), 20, 30, 50, 70 (orange), 100, 200 bp. B-E: number of CNE starts (red and green histograms) and number of bases that are part of CNEs (blue lines) B,C: counts / bp distance. D,E: counts / bp distance, normalized by the number of exon flanking sequences (partially) spanning each bin (= bases/bin * binsize). B,D: linear scale on x-axis. C,E: logarithmic scale on x-axis. Bin size: 100 bp up to kb, then 2kb

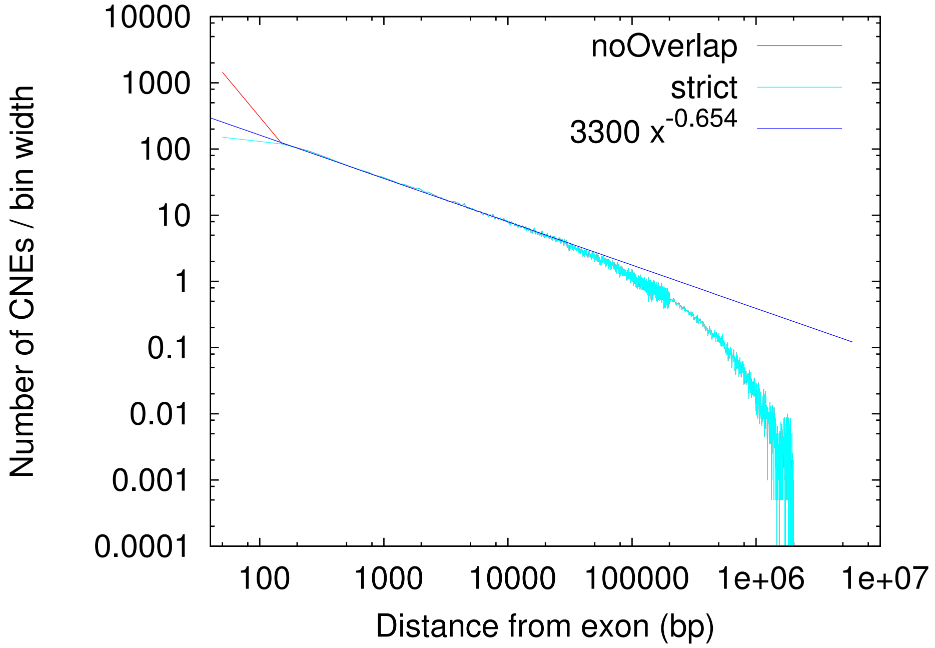

Figure S6: Distribution of distances between CNEs and their nearest exons. The blue line shows the best fitting power law for CNEs 300 bp to 30 kb away from exons. Bin width: 100 bp up to 200 kb, then 2 kb. The only difference between the "noOverlap" and "strict" CNE sets is in the first bin (<100 bp). For "noOverlap", all annotated exons are excised from the candidate elements. For "strict", all elements with any overlap with exons are removed from the set.

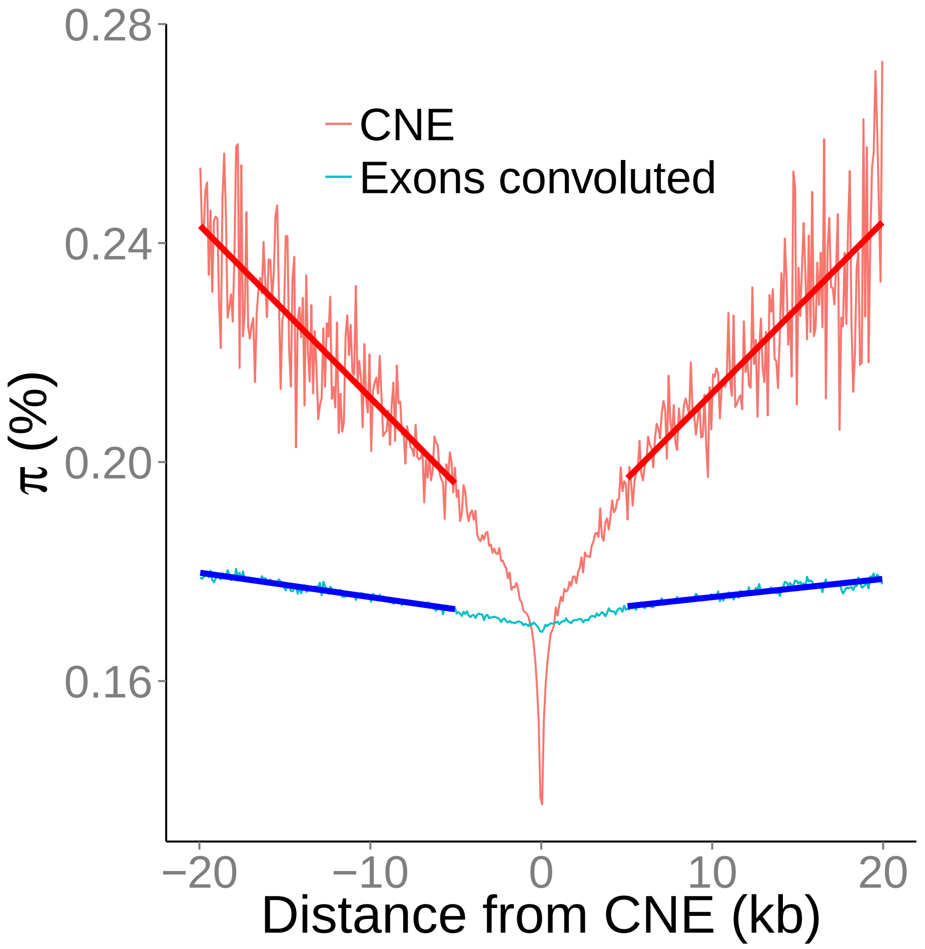

Figure S7: CNE flanks compared to convoluted exon flanks. Exon flanks were convoluted ("blurred"; see methods) using the distribution of distances between CNEs and their nearest exons. If the long length scale in the decay of the reduction of π around CNEs were entirely due to the effect of the nearest exon, the slopes far away from the CNEs are expected to be the same. Fitted slopes for the flanks at 5-20 kb from the (focal) CNE, however, show almost one order of magnitude difference: $-3.16 \pm 0.16 \times 10^{-3}$ and $3.13 \pm 0.19 \times 10^{-3}$ for CNE flanks and $-0.45 \pm 0.01 \times 10^{-3}$ and $0.33 \pm 0.01 \times 10^{-3}$ for exons.

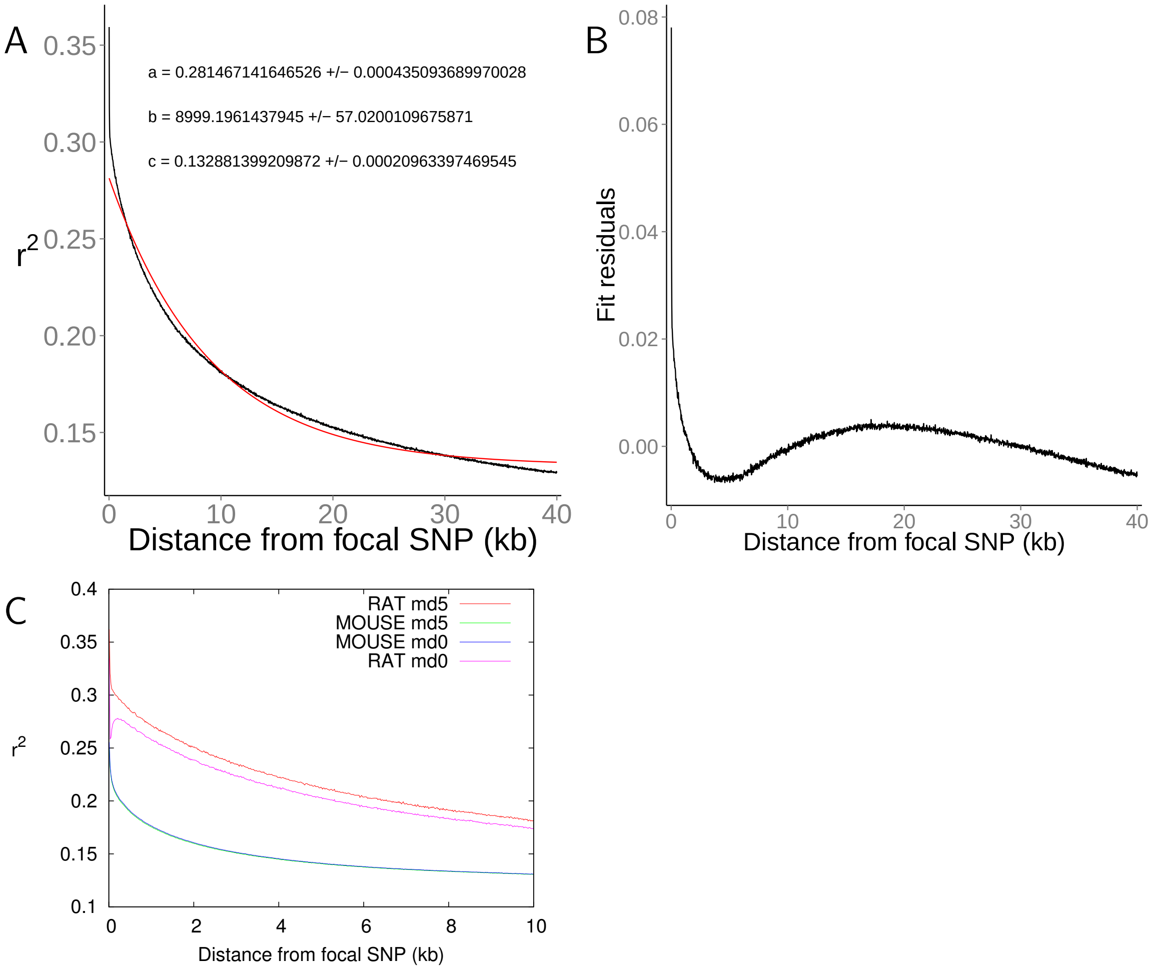

Figure S8: A: $\langle r^2 \rangle$ for all chromosomes combined, all SNPs, with offset exponential fit $f(x) = (a - c) * \exp(-x/b) + c$. B: Residuals of the fit: the decay of LD is clearly not a simple exponential process. C: for the rat data, not using the proximity filter (md=5 bp) results in a unexpected dip near SNPs. The same filter has very little effect on the mouse data.

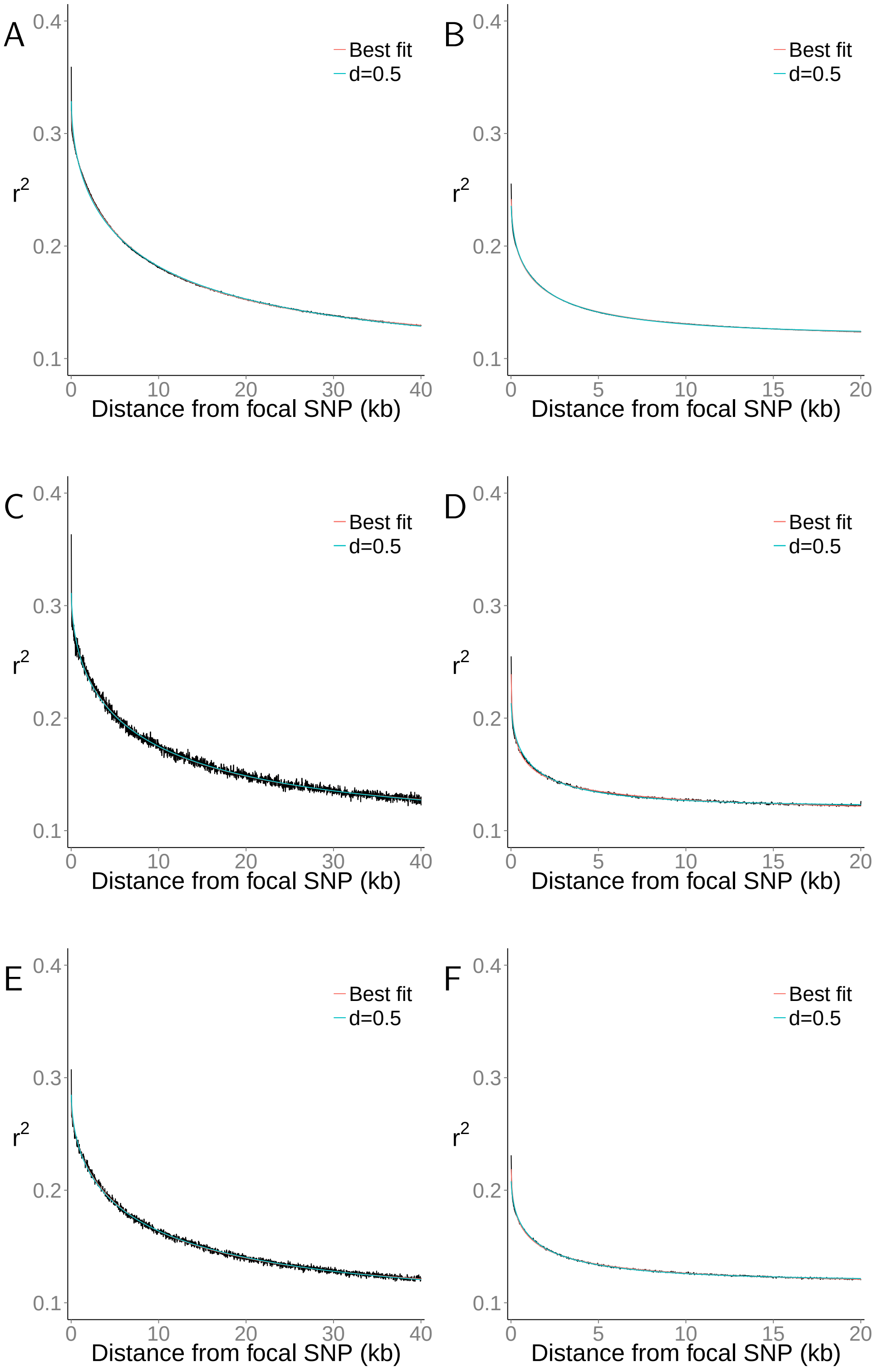

Figure S9: Stretched exponentials fit well to data of LD decay (measured by genomic $\langle r^2 \rangle$) in rat (A,C,E) and mouse (B,D,F) for all sites (A,B), focal SNPs in exons (C,D), or CNEs (E,F) only. Fitted curves are stretched exponentials of the form $g(x) = (a - c)\exp(-(x/b)^d) + c$. Best fitting curves (red) and best fitting curves with fixed d=0.5 (cyan) often overlap almost completely. Fit parameters are given in table S2

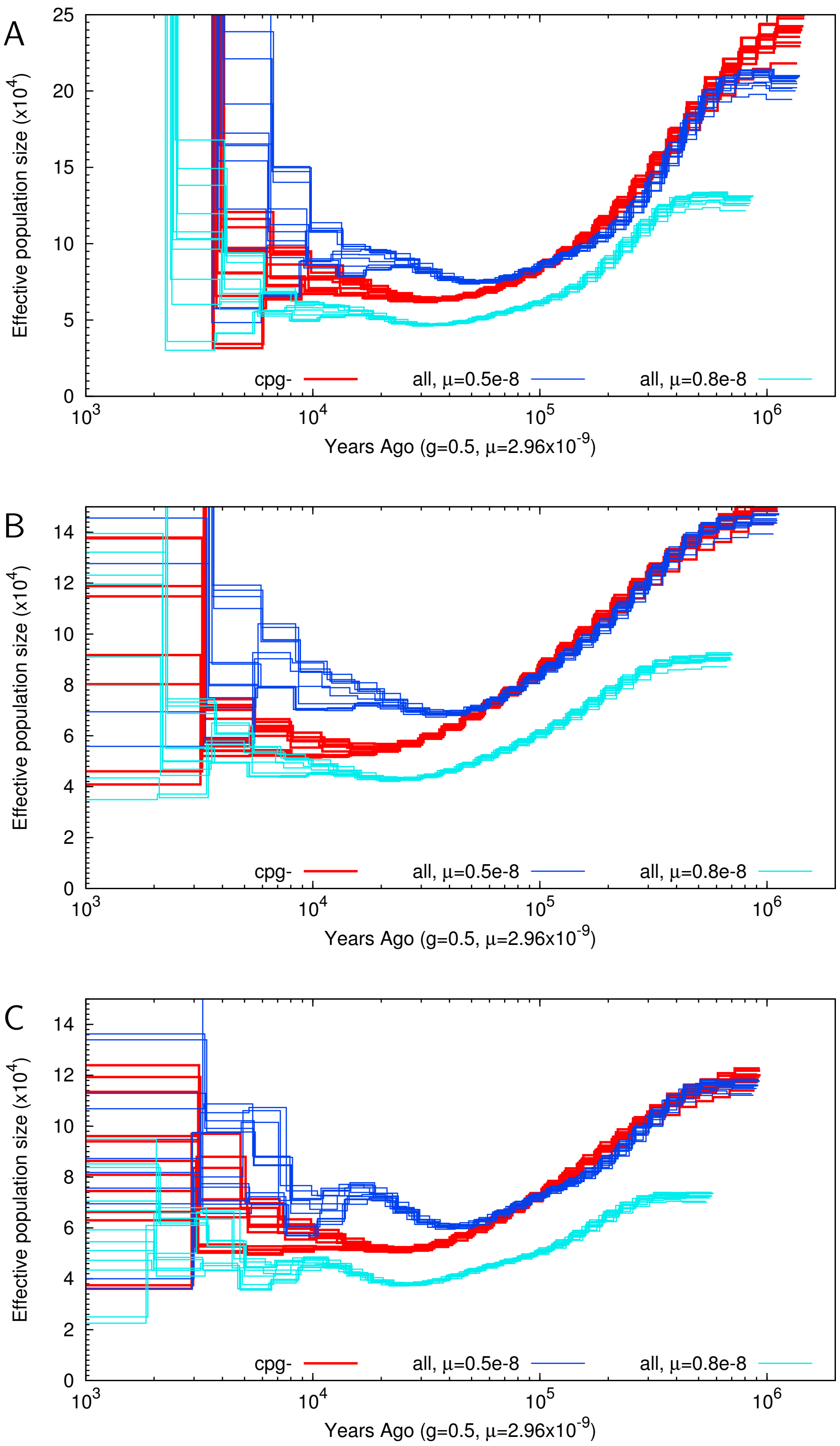

Figure S10: Inferred population history from distribution of IBS (identical by state) tract length distributions Li and Durbin (2011). Curves represent the inferred population history of individual rat samples. Estimates in red are based on non-CpG prone sites only ($\mu = 2.96 \times 10^{-9}$/bp/generation). Dark blue and cyan estimates show inferences based on all sites. Mutation rates are adapted to accomodate the higher mutation rate on CpG-prone sites. As the model assumes a homogeneous rate, which is certainly violoted, we show 2 different rates: $\mu = 5 \times 10^{-9}$/bp/generation (blue): same length of inference and $\mu = 8 \times 10^{-9}$/bp/generation (cyan): minimum of curve in the same place (∼bottleneck at the same moment). Results are shown for three values of the proximity filter parameter md: 0 bp: disable filter (A), 5 bp: default (B), 10 bp: very aggressive (C).

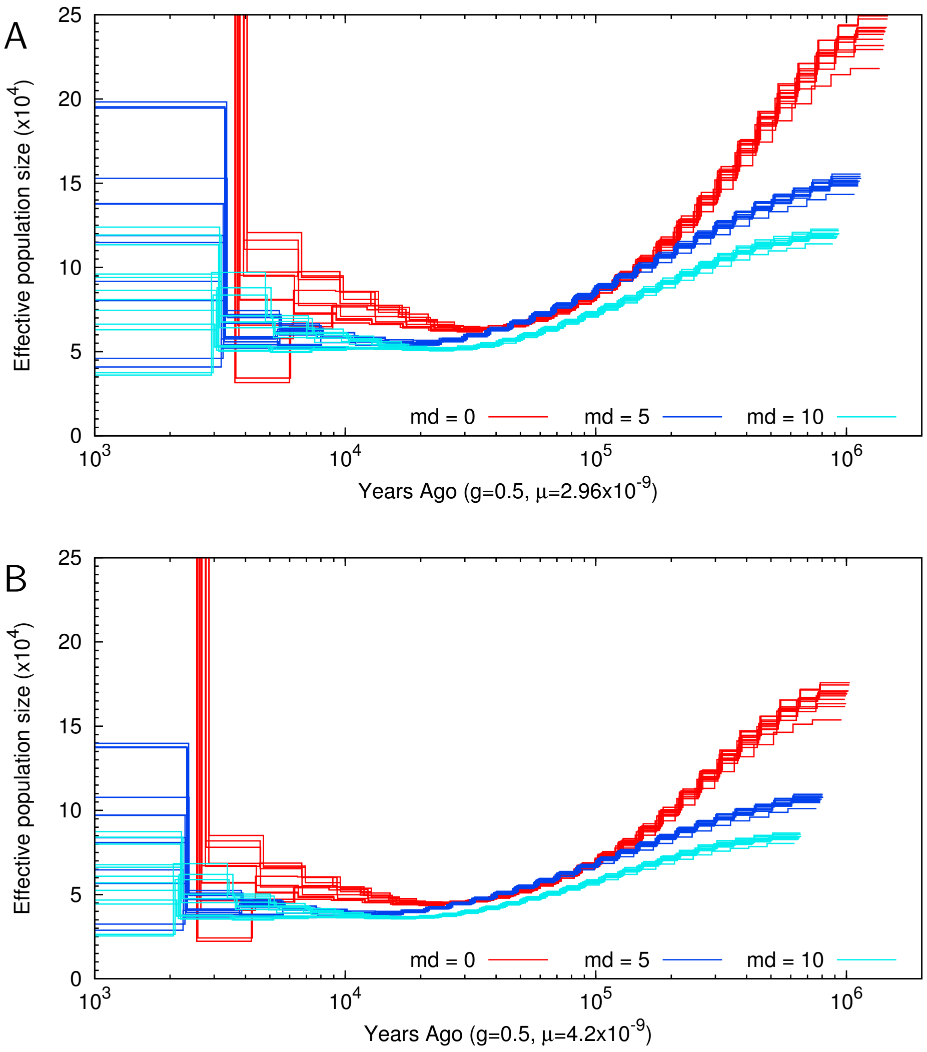

Figure S11: Inferred population history – impact of proximity filter and μ Inferred population history from distribution of IBS (identical by state) tract length distributions (Li and Durbin, 2011). Curves represent the inferred population history of individual rat samples. Different colours represent different values of the proximity filter parameter md: 0 bp: disable filter (red), 5 bp: default (blue), 10 bp: very aggressive (cyan). Estimates are based on non-CpG prone sites only and using a generation time of 0.5 year combined with a mutation rate of A: $\mu = 2.96 \times 10^{-9}$/bp/generation (our estimate based on 4-fold synonymous exonic sites), or B: $\mu = 4.2 \times 10^{-9}$/bp/generation (Ness et al., 2012).

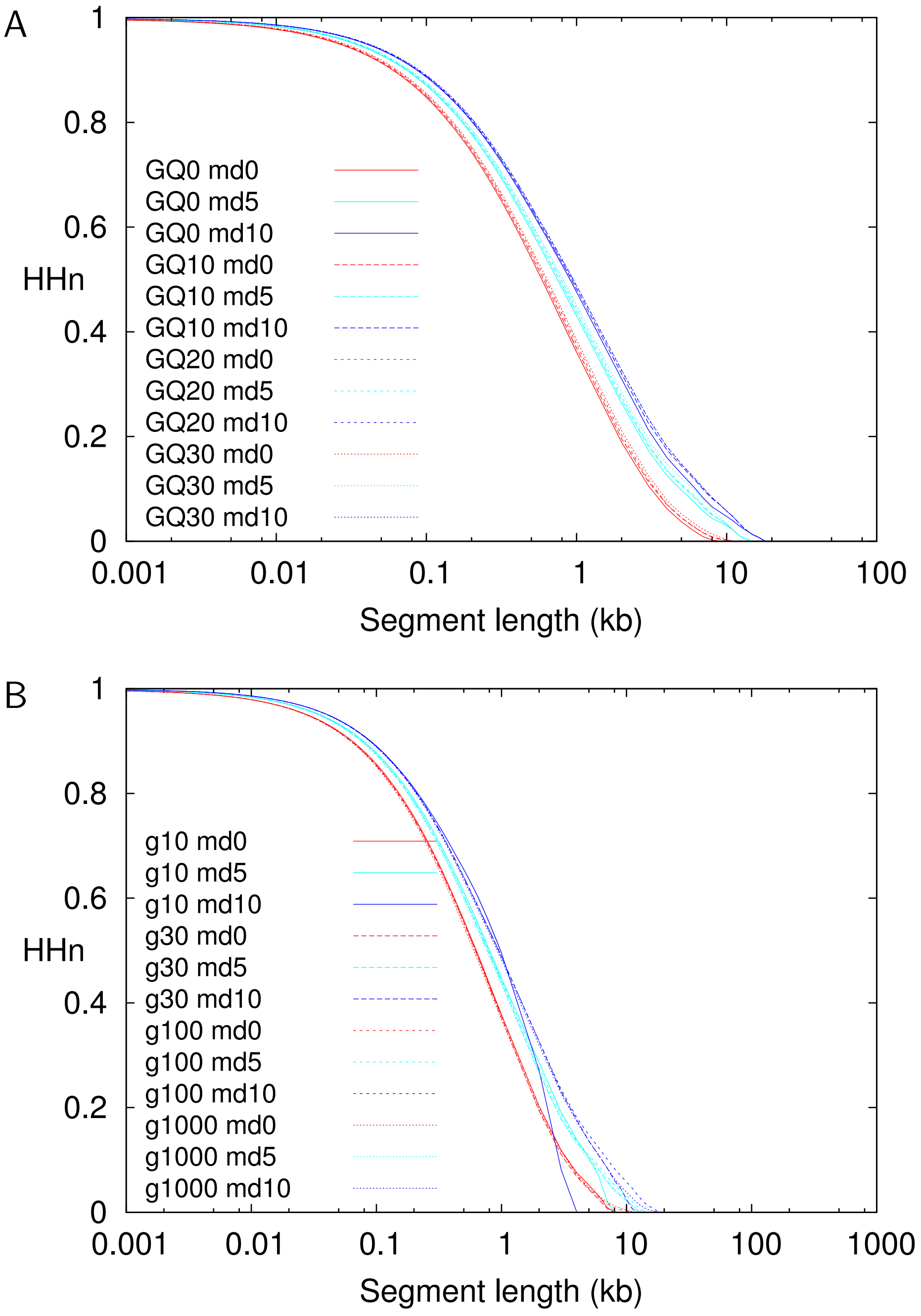

Figure S12: Relative abundance of IBS (identity by state) tracts of given length (HHn, MacLeod et al. (2013)). HHn curves were calculated on the first 1Mb of chromosome 6. A: The proximity filter parameter (md) had the largest impact and is shown with different colours: 0 bp: disable filter (red), 5 bp: default (cyan), 10 bp: very aggressive (blue). For comparison, the impact of minumum genotype call quality (GQ) is shown using different line types: 0 (solid), 10 (long dash), 20 (short dash), 30 (dotted). For the calculation we further varied bounds for normalized sample coverage (20-350% or 25-300% (default, shown), the twelve different rat samples (results shown for R3, representative example) and maximum data gap length (B: 10 (solid), 30 (long dash), 100 (short dash, used in A), 1000 bp (dotted)).

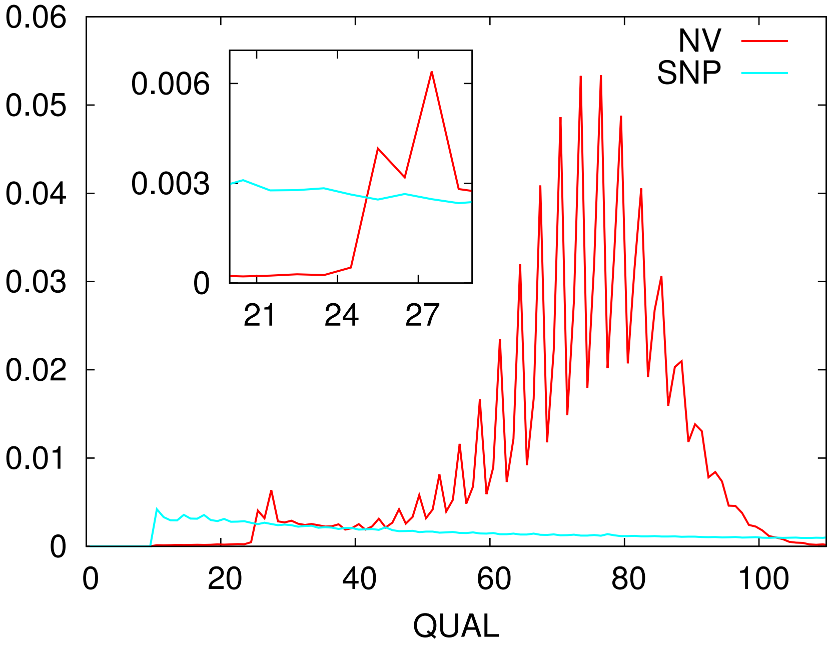

Figure S13: Histogram of phred-scaled quality (QUAL) scores for invariant (NV) and variant (SNP) sites. Note that >56% of the variant sites had a QUAL score >500. Histograms are computed over all autosomal sites. No sites with QUAL<10 are reported due to the emit cutoff used for SNP calling with GATK unified genotyper.

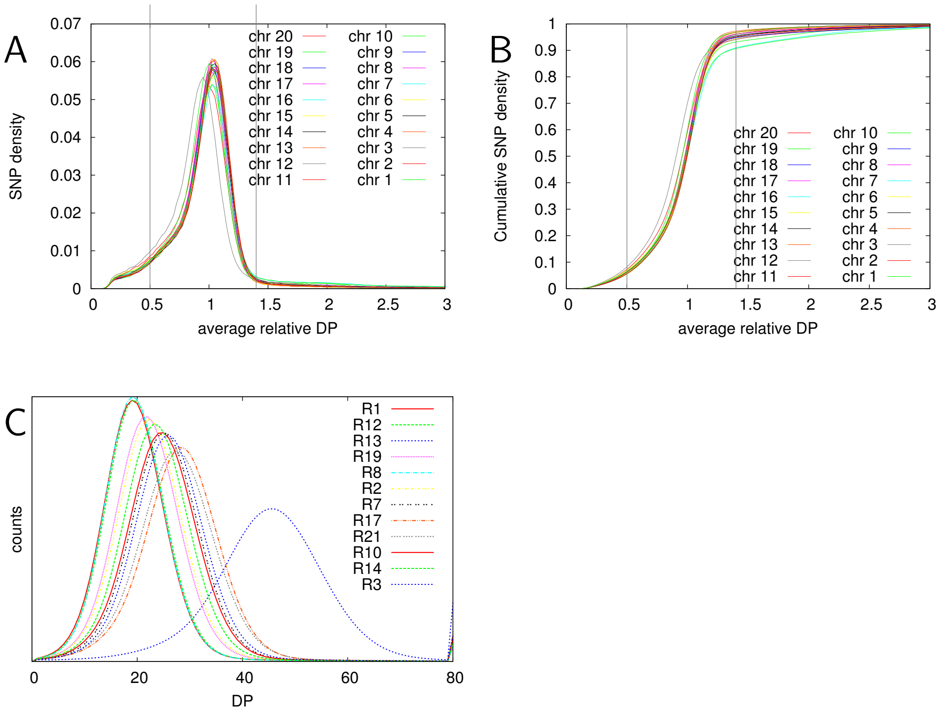

Figure S14: Average normalized DP per chromosome. A: Frequency of average relative DP over all samples, per chromosome. Note the relatively sharp bend at around 1.4. This is much sharper than for individual samples. It therefore inspired an extra DP filter. B: Cumulative frequencies. Vertical gray lines at 0.5 and 1.4. Chromosome 12 appears to be an outlier, as its modal coverage is below the autosomal mode. This chromosome has relatively high GC content. C: DP counts per sample, sum of all autosomes. A and B are calculated from all biallelic SNPs in the VCF (calculated after indel realignment), with exclusion of sites near indels (fig. 4). C is calculated from a VCF calculated using the output option "emit all sites" in GATK and a QUAL cutoff for emitting of 0.1 (to obtain maximum information in the VCF). Autosomal samples modes are the same for both VCFs.

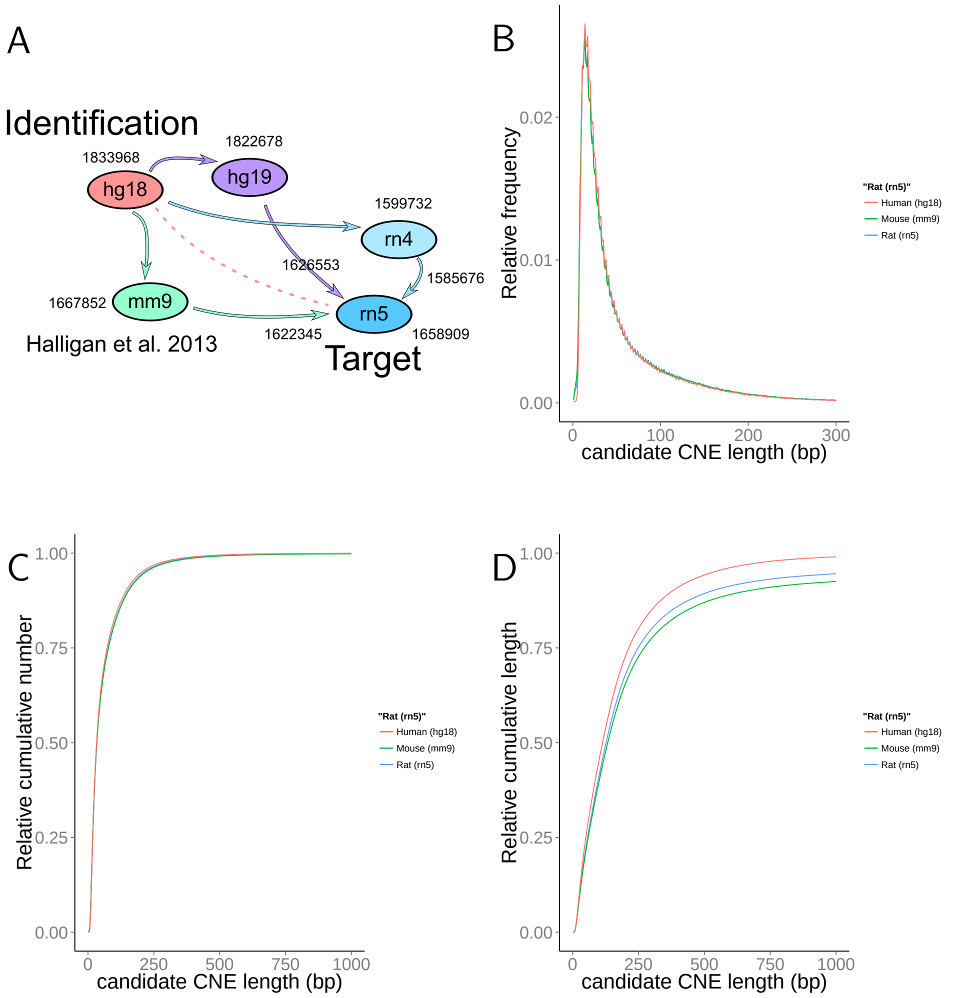

Figure S15: Characterisation of CNEs A: number of candidate CNEs, i.e., before excluding (elements that) overlap with exons, in the original set (with coordinates relative to human hg18), after liftOver to mouse mm9 (as used in Halligan et al. (2013)), at intermediate stages and in the combined set used for this manuscript. B: Histogram of CNE candidate lengths, restricted to <300 bp. C: Relative cumulative histogram of CNE candidate lengths, restricted to <1 kb. D: Relative cumulative histogram of number of bases in CNE candidates up to the specified length. The liftOver process has inflated some CNE candidates: their number is low, but they contain relatively many sites. For example: the hg18 set contains $< 1\%$ bases in CNE candidates > 1 kb, whereas the mm10 and rn5 sets contain 5-6% of such bases. This could result in an overestimate of diversity and divergence. Our current aim is to compare rat and mouse numbers, so the similarity between these two sets is desirable.

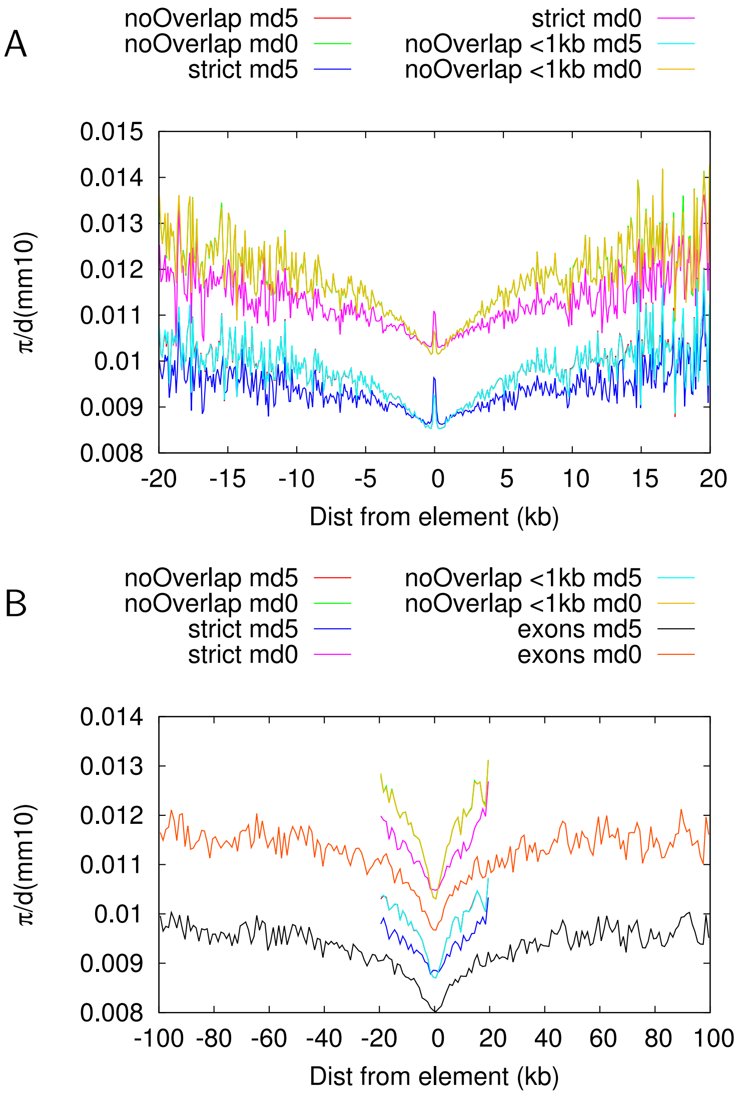

Figure S16: A: Impact of proximity filter and CNE selection rules on π/d in CNE flanks in rat (bin size: 100 bp). There is virtually no difference between the flanks of the CNE sets "noOverlap" and "noOverlap <1kb", because these sets differ only in a relatively very small number of elements. B: Comparison of π/d in CNE and exon flanks, with and without proximity filter (bin size: 1 kb).

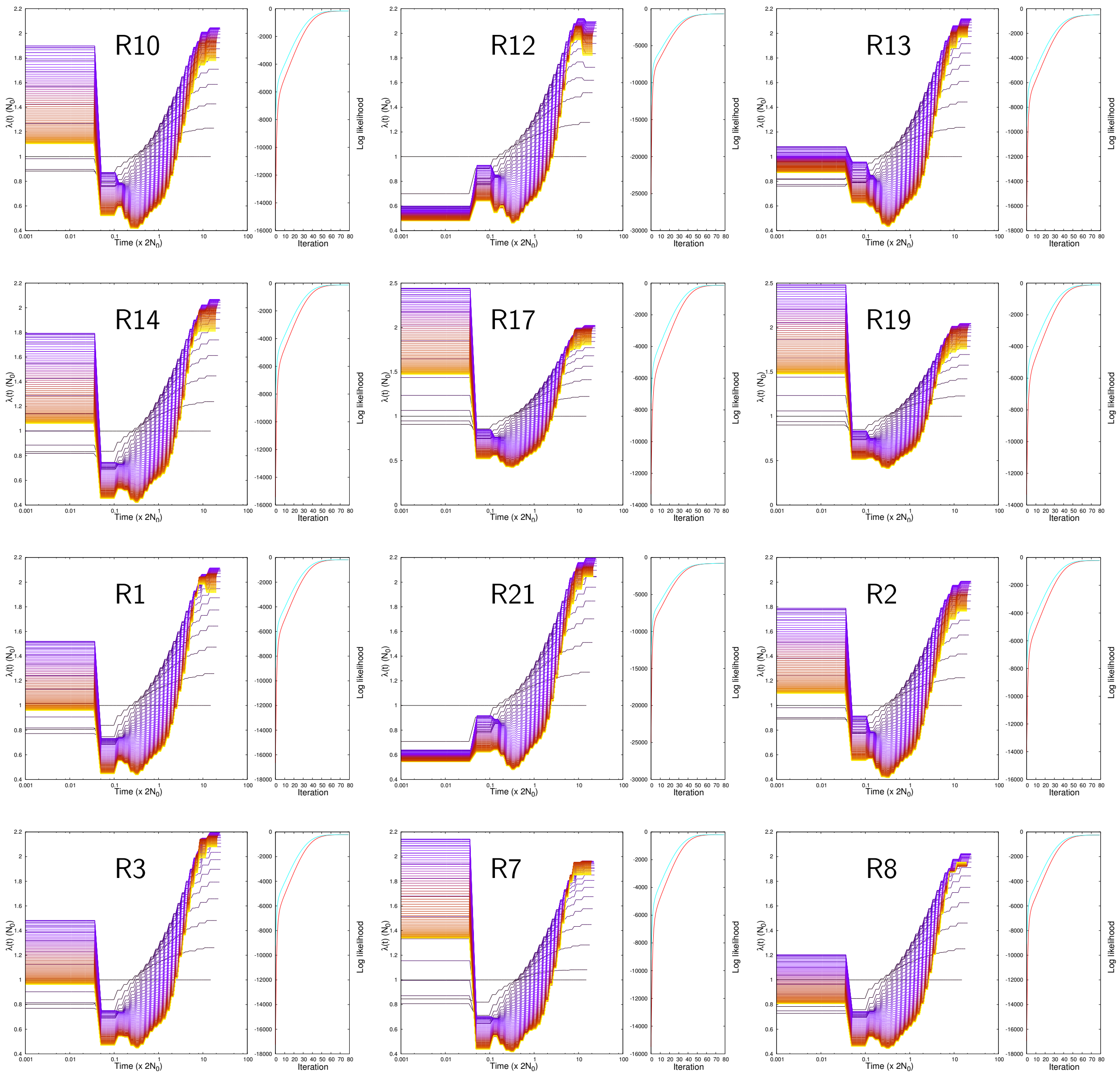

Figure S17: Convergence of history inferences of fig. 5. All 12 rats, with proximity filter (md=5), non-CpG prone sites only. Left panels show the inferred unscaled historical N_e inference for each iteration. The first iteration is coloured black, the last yellow, with purple and red inbetween. Log likelihood values from PSMC output are plotted on the right (cyan: “optimized”, red: “raw”).

	All data				No X				Ratios	
	val	2.5%	97.5%	π/d	val	2.5%	97.5%	π/d	mouse/rat	all/no X
A 0	3.107	3.048	3.167		2.976	2.920	3.036		6.578	1.044
2	10.942	10.862	11.022		10.818	10.741	10.898		6.765	1.011
C	7.086	7.020	7.152		-	-	-		6.015	-
CF	15.412	15.349	15.480		-	-	-		6.538	-
4	14.226	14.127	14.325		14.142	14.045	14.234		6.761	1.006
B 0	0.045	0.044	0.047	0.015	0.043	0.041	0.044	0.014	1.129	1.064
2	0.112	0.109	0.115	0.010	0.108	0.106	0.112	0.010	1.054	1.032
C	0.097	0.096	0.098	0.014	-	-	-	-	1.019	-
CF	0.157	0.155	0.159	0.010	-	-	-	-	1.003	-
4	0.147	0.142	0.152	0.010	0.143	0.139	0.147	0.010	1.059	1.027
C 0	0.050	0.049	0.052		0.047	0.046	0.049		1.099	1.058
2	0.119	0.117	0.122		0.116	0.114	0.119		1.041	1.027
C	0.108	0.107	0.109		-	-	-		1.013	-
CF	0.166	0.164	0.167		-	-	-		0.999	-
4	0.155	0.151	0.159		0.151	0.147	0.155		1.045	1.022
D 0	-0.428	-0.457	-0.396		-0.444	-0.476	-0.415		0.833	0.965
2	-0.271	-0.306	-0.240		-0.286	-0.318	-0.255		0.873	0.947
C	-0.410	-0.420	-0.400		-	-	-		0.951	-
CF	-0.221	-0.231	-0.211		-	-	-		0.936	-
4	-0.226	-0.263	-0.183		-0.243	-0.282	-0.205		0.840	0.931
E 0	0.472	0.463	0.483		0.456	0.447	0.465			1.036
2	1.617	1.597	1.635		1.602	1.584	1.620			1.009
C	1.178	1.166	1.189		-	-	-			-
CF	2.357	2.344	2.370		-	-	-			-
4	2.104	2.076	2.133		2.090	2.064	2.117			1.006
F 0	0.040	0.039	0.041	0.085	0.038	0.037	0.039	0.083		1.059
2	0.106	0.103	0.109	0.066	0.104	0.101	0.106	0.065		1.025
C	0.095	0.094	0.097	0.081	-	-	-	-		-
CF	0.156	0.155	0.158	0.066	-	-	-	-		-
4	0.139	0.134	0.143	0.066	0.136	0.131	0.140	0.065		1.021
G 0	0.046	0.044	0.047		0.043	0.042	0.045			1.053
2	0.115	0.112	0.117		0.112	0.110	0.115			1.022
C	0.107	0.105	0.108		-	-	-			-
CF	0.166	0.164	0.167		-	-	-			-
4	0.148	0.144	0.152		0.146	0.142	0.149			1.018
H 0	-0.514	-0.535	-0.473		-0.531	-0.564	-0.501			0.969
2	-0.310	-0.347	-0.280		-0.319	-0.352	-0.288			0.973
C	-0.432	-0.441	-0.421		-	-	-			-
CF	-0.236	-0.245	-0.227		-	-	-			-
4	-0.269	-0.304	-0.223		-0.282	-0.322	-0.243			0.957

Table S1: Diversity, divergence, Tajima's D, including all data of fig. 1. Within each sub-table, values are given for 0-fold degenerate exonic sites (0), 2-fold (2), CNEs (C), CNE neutral standard (CF) and 4-fold (4). Statistics: Jukes Cantor corrected divergence (A,E), θ_π (B,F), Watterson's θ_W (C,G), Tajima's D (D,H). All statistics are calculated on the non-CpG prone bases that have corresponding mm10 (A-D) or *R. rattus* (E-H) bases, respectively. No X: excluding non-canonical transcripts (i.e., not appearing in eggNog database). "Val" values are calculated using each relevant base exactly once. Confidence intervals for exonic sites are based on the "NoOv" (all data) and "all\X" sets, respectively. For CNEs and CNE neutral standard, the genome was subdivided in non-overlapping 1 Mb blocks. All based on 1000 bootstrapping replicates.

A

focal SNPs	a	b	c	d	Residuals
mouse exon	0.3234 ± 0.0065	174.8 ± 20.3	0.1119 ± 0.0006	0.2364 ± 0.0068	0.001191139
mouse cne	0.2413 ± 0.0012	846.1 ± 20.1	0.1156 ± 0.0002	0.3657 ± 0.0045	0.0005138185
mouse all	0.2574 ± 0.0007	1295 ± 13.9	0.1187 ± 0.0001	0.4366 ± 0.0031	0.0003936348
rat exon	0.3128 ± 0.0014	8083 ± 105	0.1080 ± 0.0010	0.5331 ± 0.0075	0.01871628
rat CNE (NO)	0.2819 ± 0.0007	8309 ± 63.6	0.1056 ± 0.0005	0.5640 ± 0.0049	0.006267381
rat CNE (S)	0.2841 ± 0.0007	7932 ± 60.1	0.1060 ± 0.0005	0.5625 ± 0.0049	0.006608745
rat all	0.3245 ± 0.0005	8435 ± 33.6	0.1106 ± 0.0003	0.5679 ± 0.0026	0.002535309

B

focal SNPs	a	b	c	Residuals
mouse exon	0.2223 ± 0.0005	1149 ± 18.9	0.1216 ± 0.0001	0.002766982
mouse cne	0.2158 ± 0.0004	1360 ± 13.7	0.1195 ± 0.0001	0.0009167815
mouse all	0.2454 ± 0.0003	1521 ± 9.3	0.1210 ± 0.0001	0.0005450216
rat exon	0.3186 ± 0.0006	8206 ± 117	0.1036 ± 0.0005	0.0188766
rat CNE (NO)	0.2913 ± 0.0004	8584 ± 83.4	0.0982 ± 0.0003	0.006768374
rat CNE (S)	0.2936 ± 0.0004	8111 ± 78.1	0.0991 ± 0.0003	0.007111008
rat all	0.3365 ± 0.0003	8766 ± 49.9	0.1009 ± 0.0002	0.003384385

Table S2: Fit parameters for fitting a stretched exponential function to LD ($\langle r^2 \rangle$) decay with physical distance from focal SNPs. Model: $g(x) = (a-c)\exp(-(x/b)^d) + c$. A: Fitting all parameters simultaneously. B: With fixed stretch exponent $d = 0.5$. All fits were obtained using nonlinear least squares (nls) from the R statistical package. Data for mouse: 1000 bins of 20 bp up to 20 kb, no proximity filter applied (md=0; almost no difference with md=5, see fig. S8C). Data for rat: 2000 bins of 20 bp up to 40 kb, using proximity filter (md=5). CNE (NO): "No overlap" selection rule for CNEs, following Halligan et al. (2013). CNE (S): "Strict" selection rule for CNEs. See methods for details on selection rules.

Sample	Name	DP
“ERS215788”	*R. rattus*	33
“ERS215789”	R1	19
“ERS215790”	R12	19
“ERS215791”	R13	46
“ERS215792”	R19	22
“ERS215793”	R8	19
“ERS215794”	R2	22
“ERS215795”	R7	25
“ERS215796”	R17	28
“ERS215797”	R21	27
“ERS215798”	R10	25
“ERS215799”	R14	23
“ERS215800”	R3	26

Table S3: Coverage statistics. Names of samples and modal autosomal coverage (DP).

References

Eyre-Walker A and Keightley PD, 2009. Estimating the rate of adaptive molecular evolution in the presence of slightly deleterious mutations and population size change. *Mol Biol Evol*, **26**(9):2097–2108.

Halligan DL, Kousathanas A, Ness RW, Harr B, Ery L, Keane TM, Adams DJ, and Keightley PD, 2013. Contributions of protein-coding and regulatory change to adaptive molecular evolution in murid rodents. *PLoS Genet*, **9**(12):e1003995.

Li H and Durbin R, 2011. Inference of human population history from individual whole-genome sequences. *Nature*, **475**(7357):493–496.

MacLeod IM, Larkin DM, Lewin HA, Hayes BJ, and Goddard ME, 2013. Inferring demography from runs of homozygosity in whole-genome sequence, with correction for sequence errors. *Mol Biol Evol*, **30**(9):2209–2223.

Ness RW, Zhang Y-H, Cong L, Wang Y, Zhang J-X, and Keightley PD, 2012. Nuclear gene variation in wild brown rats. *G3 (Bethesda)*, **2**(12):1661–1664.

Weir BS, 1979. Inferences about linkage disequilibrium. *Biometrics*, :235–254.
